# Detection of Congenital Toxoplasmosis and Cytomegalovirus Infections Using Paired Sample Serodiagnosis from Suspected Cases at a Tertiary Teaching Hospital in Malaysia

**DOI:** 10.21315/mjms2024.31.4.8

**Published:** 2024-08-27

**Authors:** Abdirahman Hussein Elmi, Ahmad Adebayo Irekeola, Muhammad Amiruddin Abdullah, Zeehaida Mohamed

**Affiliations:** 1 Department of Medical Microbiology and Parasitology, School of Medical Sciences, Universiti Sains Malaysia, Kelantan, Malaysia; 2 Hospital Universiti Sains Malaysia, Kelantan, Malaysia; 3 Department of Medical Laboratory Sciences, Faculty of Medicine and Health Sciences, Jamhuriya University of Science and Technology, Mogadishu, Somalia

**Keywords:** congenital, toxoplasmosis, cytomegalovirus, serodiagnosis, Malaysia, paired sample

## Abstract

**Background:**

Congenital toxoplasmosis and congenital cytomegalovirus (cCMV) infections are noteworthy in Malaysia and can cause serious health problems in neonates. The prompt and effective detection and treatment related to both illnesses may mitigate the possibility of adverse consequences from both infections.

**Methods:**

A total of 219 neonates with suspected clinical indications of congenital toxoplasmosis and/or cCMV infections from January 2022 to December 2022 were enrolled. The first samples for IgM and IgG antibodies were screened by electrochemiluminescence immunoassay. For positive results indicative of congenital toxoplasmosis and cCMV infections, second serum samples were requested and tested within a period of 2–4 weeks after testing the first sample.

**Results:**

From the 219 first serum samples, the overall seroprevalence of congenital toxoplasmosis antibodies in suspected cases was 53%; meanwhile, the overall seroprevalence of cCMV in the suspected cases was 98.6%. The results of the paired serum sample collected for investigating congenital toxoplasmosis cases revealed that 47% of the cases presented no serological evidence of exposure while the remaining 53% of cases might have acquired passive immunity from the mother. For cCMV, the number of cases with no serological evidence of exposure was 1.4%, whereas acute infection was 1.8% and possible passive immunity from the mother represented 96.8%.

**Conclusion:**

This study found a high seroprevalence of congenital toxoplasmosis and cCMV infections, probably because they are suspected cases. This study also indicates that using paired sample analysis in the categorisation of cases can aid in accurate diagnosis and more effective treatment.

## Introduction

Toxoplasmosis and cytomegalovirus (CMV) are prevalent pathogens that can be vertically transmitted to the developing foetus. The clinical presentations of many congenital diseases show similarities, rendering it challenging to determine an accurate etiological component through clinical examination alone (1). Congenital toxoplasmosis and congenital cytomegalovirus (cCMV) infections are common congenital illnesses in Malaysia that have the potential to cause serious complications in neonates (2, 3). These infections possess different aetiologies.

*Toxoplasma gondii* is a parasitic organism responsible for causing the zoonotic disease recognised as toxoplasmosis (4). Toxoplasmosis is a possible complication during pregnancy that can result in significant neurological or ocular damage or even mortality in neonates (5). Previous studies have documented the occurrence of toxoplasmosis in Malaysia. The overall seroprevalence (IgG or IgM alone, or both) of toxoplasmosis ranges from 1%–65% (2, 6–12).

CMV is a member of the β-herpesviridae subfamily and is notable for its propensity to induce pathology in neonates who acquire the infection congenitally (13). cCMV infection is widespread globally, making it the most prevalent congenital viral infection. It is also a primary contributor to hearing impairment and neurological disorders in children (14). The reported prevalence of cCMV in Malaysia has consistently exceeded 90% in previous studies (15, 16).

The seroprevalence of congenital toxoplasma and cCMV among neonates has several aspects and can be influenced by various factors, including the mother’s age, socio-economic status, access to sufficient prenatal care and geographical location (17).

In general, standard serological assays are used for the diagnosis of congenital toxoplasmosis and cCMV infection. The tests aim to detect the presence of *Toxoplasma* (Toxo)-specific IgM and IgG antibodies, as well as CMV-specific IgG and IgM antibodies. These tests can be carried out separately or as components of a TORCH (*Toxoplasma*, others [syphilis], rubella, CMV and herpes simplex virus) titer panel (18). Performing a serological test on a neonatal sample for the first time alone does not provide sufficient evidence to classify clinical cases between an acute infection in the newborn and possible passive immunity acquired from the mother. As a result, potential misdiagnosis and administration of inappropriate treatment may occur. Thus, the aim of this study was to determine the prevalence of congenital toxoplasmosis and cCMV infections using paired sample serodiagnosis and to evaluate the association with the clinical manifestations of the infections. The study also attempts to distinguish the possible passive immunity (acquired from the mother) from the active immunity of the neonates.

## Methods

### Study Subjects (Population Description)

Of the 9,417 documented births at HUSM, the current study assessed a total of 219 neonates who presented with clinically suspected cases of congenital toxoplasmosis and cCMV infections. These neonates were born at Hospital Universiti Sains Malaysia (HUSM) during the period spanning from January 2022 to December 2022.

### Criteria for Study Enrolment

**Figure 1:**
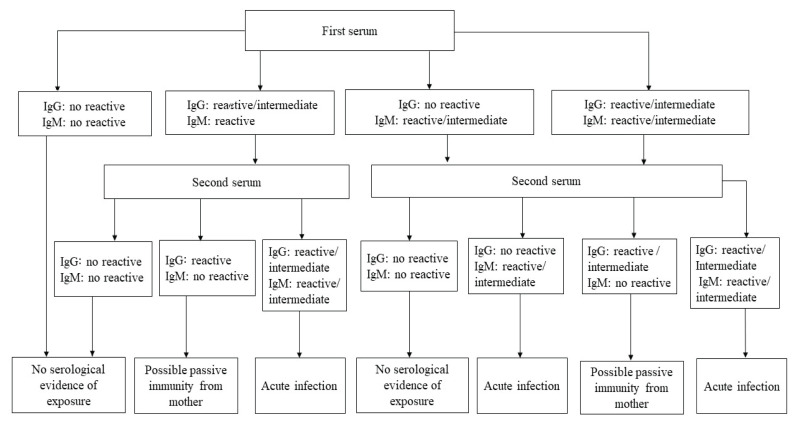
Serodiagnosis algorithm for toxoplasmosis and CMV infection in neonates

Newborns exhibiting IgM and IgG antibody titer indicative of congenital toxoplasmosis and cCMV infections were requested to furnish a second serum sample within a period of 2–4 weeks. The serological profiles of the 219 cases were examined and correlated with the clinical data retrieved from neonates’ records for the purposes of diagnosis and infection classification. The exclusion criteria employed in the study comprised neonates who were over 30 days old and did not satisfy the criteria for a paired sample in the case of congenital toxoplasmosis and cCMV infections, which required a first serum sample. Additionally, neonates who passed away after the first serum sample collection and those whose second serum sample was procured over a period of 4 weeks were also excluded. The study employed a diagnostic approach, as depicted in Figure 1.

### Collection of Samples and Laboratory Examination

Serum of blood collected from the neonates were serologically screened for anti-*Toxoplasma* IgM (anti-Toxo IgM) and IgG and anti-CMV IgM and IgG using elecsys *Toxoplasma*, and CMV IgG and IgM immunoassays (Roche, Germany) in accordance with the manufacturer’s instructions. The test was fully automated and the detection of Toxo IgG and CMV IgG was based on the sandwich principle. The total duration of the assay was 18 min and involved two incubation steps. In the first incubation, 20 uL of the sample, biotinylated recombinant Toxo- or CMV-specific antigens and Toxo- or CMV-specific recombinant antigens labeled with a ruthenium complex form a sandwich complex. In the second incubation, after the addition of streptavidin-coated microparticles, the complex binds to the solid phase via the interaction of biotin and streptavidin.

The reaction mixture was aspirated into the measuring cell, where the microparticles were magnetically captured onto the surface of the electrode. Unbound substances were then removed with ProCell/ProCell M. Application of a voltage to the electrode induced chemiluminescent emission, which was measured by a photomultiplier. Results were determined via a calibration curve, which was instrument-specifically generated by 2-point calibration and a master curve provided via the reagent barcode or e-barcode.

The Toxo IgG and CMV IgG results were expressed as international unit (IU) and unit (U), respectively. Based on the kit interpretation, sera with IgG titer of < 1 IU/mL were considered non-reactive for anti-*T. gondii* IgG, whereas sera with IgG titer of ≥ 1 IU/mL– < 30 IU/mL and ≥ 30 IU/mL were considered indeterminate and reactive, respectively. For anti-CMV sera with IgG titer of < 0.5 U/mL were reported as non-reactive. While IgG titers of 0.5 U/mL– < 1.0 U/mL were reported as indeterminate and IgG titers of ≥ 1 U/mL were reported as reactive.

The test for IgM detection was also fully automated and based on the μ-Capture test principle. The total duration of the assay was 19 min. In the first incubation, 10 μL of the sample was automatically prediluted to 1:20 with diluent Universal (Roche, Germany). For *T. gondii* detection, *T. gondii*-specific recombinant antigen labeled with a ruthenium complex was added. Anti-Toxo IgM antibodies present in the sample reacted with ruthenium-labeled *T. gondii*-specific recombinant antigen. Meanwhile, for CMV detection, CMV biotinylated monoclonal anti-h-IgM-specific antibodies were added.

During the second incubation, biotinylated monoclonal h-IgM-specific antibodies and streptavidin-coated microparticles were added for *T. gondii* detection. The complex binds to the solid phase via the interaction of biotin and streptavidin. Meanwhile, for CMV detection, CMV-specific recombinant antigens labeled with ruthenium complex and streptavidin-coated microparticles were added. Anti-CMV IgM antibodies present in the sample react with the ruthenium-labeled CMV-specific recombinant antigen. The complex binds to the solid phase via the interaction of biotin and streptavidin. The IgM reaction mixture was aspirated into a measuring cell where the microparticles were magnetically captured onto the surface of the electrode. Unbound substances were then removed with ProCell/ProCell M. Application of a voltage to the electrode then induced chemiluminescent emission, which was measured by a photomultiplier. Results were then determined automatically by the software.

Toxo IgM and CMV IgM results were expressed through the cut-off index (COI - sample signal/cut-off). Toxo IgM samples with COI of < 0.8 were classified as non-reactive. Samples with COI of ≥ 0.8 and < 1.0 were classified as indeterminate, whereas samples with a COI of ≥ 1.0 were considered IgM reactive. CMV IgM results were also reported in the COI. Samples for CMV IgM with a COI of < 0.7, ≥ 0.7 but < 1.0 and ≥ 1.0 were reported as non-reactive, indeterminate and reactive, respectively.

### Infection Classification and Data Interpretation

The sera underwent first-sample screening for the presence of anti-Toxo IgM and IgG, CMV IgM and IgG, and suspected clinical symptoms. Second serum samples of congenital *toxoplasmosis* and cCMV were obtained from neonates who had previously provided the first serum samples, with a time interval of 2–4 weeks between collections. Based on the serological test performed on the paired serum sample, the results were classified as no serological evidence of exposure, acute infection and possible passive immunity from the mother.

### Statistical Analysis

The data were gathered and subsequently analysed utilising the SPSS Statistics software version 27.0. The utilisation of descriptive statistics was implemented to analyse the data related to the dependent variables and identify the prevalence of congenital toxoplasmosis and cCMV infections among neonates. The Pearson’s chi-squared test and Fisher’s exact test were applied to assess the distribution of outcomes related to congenital toxoplasmosis and cCMV infections across various clinical presentations.

## Results

The screening outcomes of the first and second serum samples were solicited with an interval of 2–4 weeks for congenital toxoplasmosis and CMV infections. The suspected cases underwent an assessment to determine the presence of antibodies, specifically congenital toxoplasmosis IgM and IgG and cCMV IgM and IgG. Patients who met the inclusion criteria were selected for subsequent analysis and their medical records were tracked. The infection was categorised as either no serological evidence of exposure, acute infection or possible passive immunity from the mother based on an analysis of the serological profiles of the paired serum samples (Figure 1).

### Initial Serological Findings

**Figure 2:**
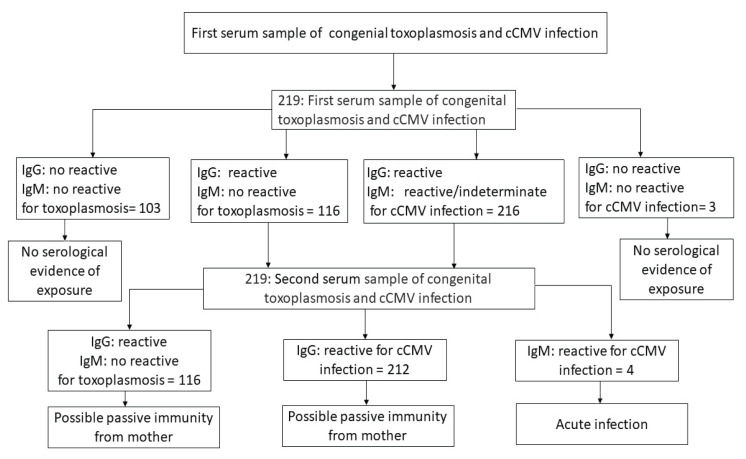
Conceptual framework case selection results

A total of 219 first serum samples from congenital toxoplasmosis IgG screening findings revealed that the overall seroprevalence of congenital toxoplasmosis antibodies in suspected cases was 116 (53%), with no IgM-positive samples. Meanwhile, IgG and IgM seropositive cases were found for neonatal cCMV infection, with an overall seroprevalence (IgG or IgM alone, or both) of 216 (98.6%) (Figure 2).

### Paired-Serum Analysis

Additionally, the results of the first and second serum samples collected to investigate cases of congenital toxoplasmosis infection classified a total of 103 cases (47%) as having no serological evidence of exposure and 116 cases (53%) as having possible passive immunity from the mother. According to the results of cCMV, the number of cases with no serological evidence of exposure was 3 (1.4%), whereas acute infection was 4 (1.8%) and possible passive immunity from the mother was 212 (96.8%) (Figure 2).

### Demographic and Clinical Characteristics of the Neonates

An overview of the clinical presentation of neonate patients who were born with congenital toxoplasmosis and cCMV infection shows that the small for gestation age (SGA) was represented by 59.8%, as shown in Table 1. The demographic distribution of the patients was also assessed. Based on ethnicity, most of the patients were Malay (*n* = 114 [95.8%]), followed by Chinese (*n* = 3 [2.5%]) and others (*n* = 2 [1.7%]). Gender distribution revealed that 113 (51.6%) were females and 106 (48.4%) were males.

The clinical presentation and interpretation of the outcome of congenital toxoplasmosis are provided in Table 2. A statistically significant link was found between SGA and the outcome of congenital toxoplasmosis (*P* = 0.021), but there was no link between the remaining clinical presentation and the interpretation of the outcome of congenital toxoplasmosis. In addition, the clinical manifestation and analysis of the findings of cCMV infection are presented in Table 3. Based on the statistical analysis, there was no observed correlation between the clinical presentation of cCMV and the interpretation of the outcomes.

## Discussion

Congenital infections caused by *T. gondii* and CMV exhibit similar clinical manifestations. Early detection and treatment can help alleviate the potential long-term effects associated with both infections. The incidence of coinfection is infrequently documented (19). The findings of the current study of first serum revealed that the overall incidence of congenital toxoplasmosis was 53%, as demonstrated by IgG-positive cases. This study showed an increased prevalence compared to the previous studies of Mohamed and Hajissa (11) and Thangarajah et al. (2) conducted at the Hospital Universiti Sains Malaysia, which documented rates of 44.2% and 38%, respectively. Nevertheless, it is noteworthy that the study conducted by Mohamed and Hajissa did not differentiate the cases into possible passive immunity from mothers or acute cases (11). On the other hand, Thangarajah et al. (2) distinguished clinical cases into groups. Importantly, a diagnostic algorithm utilising paired serum samples allowed further categorisation of the disease, a crucial aspect for the commencement of treatment.

Furthermore, our study on congenital toxoplasmosis was categorised into two groups based on paired sample serological analysis: cases with no serological evidence and cases with passive immunity from the mother. Out of the total cases, 47% had no serological evidence of exposure, while 53% had possible passive immunity from the mother. According to the study by Thangarajah and co-workers (2), a clinical classification was done for the paired samples of 141 newborns and infants. The findings revealed that 93.6% of cases were attributed to passive immunity from the mother, 2.1% were associated with acute infection and 4.3% were indicative of possible congenital infection (2).

To assess the cases of possible passive immunity from mother, it is recommended to obtain serum from the mother and analyse serologically. The diagnosis of acute congenital toxoplasmosis can be achieved by detecting serological antibodies for anti-*T. gondii*-specific IgM or by demonstrating a significant rise in specific IgG antibody titer in paired serum samples (20). It is important to note that IgM antibodies can persist in neonates or infants of 1 month–6 months of age (21). Additionally, to confirm acute infection, it is suggested to apply molecular detection, such as PCR, in combination with other diagnostic techniques. This approach has considerably enhanced the prenatal diagnosis of congenital toxoplasmosis (22). Nonetheless, this present study did not observe any documented IgM antibody indicative of acute infection in congenital toxoplasmosis. Based on the first serum analysis, this study found an overall seroprevalence of cCMV infection of 98.6%, which was higher than that of congenital toxoplasmosis. The high seroprevalence observed is similar to the findings of Hamid et al. (16), who reported a cCMV infection rate of 99.3% from the first serum samples of infants at the same hospital.

From the analysis of the paired serum samples, three categories of cCMV cases were established in this study: no serological evidence of exposure, acute infection and possible passive immunity from the mother. The overall percentage of cases with no serological evidence of exposure was 1.4%, whereas acute infection and possible passive immunity from the mother represented 1.8% and 96.8%, respectively. According to the study by Hamid et al. (16), who used paired serum samples for serodiagnosis and PCR, three categories of cCMV infection were established: i) acute CMV infection (21.4%), ii) passive immunity (57.6%) and iii) inconclusive (20.9%). The results of our study show that the percentage of possible passive immunity from the mother was 96.8%. This high prevalence could be due to the fact that a significant percentage of children infected by cCMV were delivered by women who exhibited positive results for CMV IgG antibody testing. In order to further the understanding of this scenario, it is necessary to determine maternal passive immunity by concurrently obtaining serum from a mother for a serological or molecular test to verify the presence of an actual infection. The standard microbiological diagnosis protocol in Malaysia does not encompass routine CMV screening for neonates and pregnant women. Screening for CMV infection is typically performed only in cases where there is a suspicion of such an infection (16, 23).

Further, the outcomes of our studies demonstrate that the incidence of acute infection cases was 1.8%, which matches the findings reported by Hamid et al. (16) in the same hospital, especially in terms of serology IgM positivity (1.5%) among infants. The detection of IgM antibodies has been employed as an indicator of the presence of a recent or acute infection (24). The standard practice involves promoting the utilisation of serology in conjunction with PCR correlation. However, the financial implications and the lack of PCR equipment for CMV screening necessitate the use of serological tests alone in countries with limited resources (23, 25).

The clinical manifestations assessed in this study demonstrated that congenital toxoplasmosis and cCMV infection may exhibit overlapping signs and symptoms, and a neonate may present with a concomitant occurrence of 2–3 clinical manifestations. The overall clinical observations indicated that a significant proportion of cases were classified as SGA (59.8%). Other notable findings included neonatal jaundice (5.5%), sepsis (2.7%), presumed sepsis (2.7%), premature birth (1.4%), and cases of fever with rashes and intrauterine growth restriction (IUGR) (0.9% each). Our study found no significant correlation between the clinical presentations assessed and the outcome of congenital toxoplasmosis, except for the SGA symptom, which showed a high level of significance (*P* = 0.021). A prevalence of 52.6% was observed for SGA in cases where passive immunity was acquired from the mother. On the other hand, the clinical manifestation and the cCMV result did not demonstrate any significant association. This observation could be associated with the concept that congenital toxoplasma and cCMV show similar clinical manifestations and diagnostic challenges (19).

The limitation of this study is the absence of clarification regarding the initiation of treatment. Beyond that, it should be noted that the study’s data collection period was limited, and as such, the mother’s serum, including IgG levels resulting from passive immunity from either the mother or neonate and long-term follow-up to assess symptom development and seroconversion, was not incorporated.

## Conclusion

This study found a high seroprevalence of congenital toxoplasmosis (53%) and cCMV infections (98.6%) from the first serum samples analysis. This study also indicates that using paired sample analysis in the categorisation of cases can aid in accurate diagnosis and more effective treatment, thereby reducing the likelihood of misdiagnosis. The classification of infection cases typically requires 2–4 weeks using serology identification methods. In order to facilitate future study efforts, it is suggested that researchers explore cost-effective strategies to detect congenital toxoplasmosis and cCMV infections, thereby enabling rapid detection and treatment.

## Figures and Tables

**Table 1:** Clinical signs and symptoms of congenital toxoplasmosis and cCMV infections

Clinical presentation	*n* (%)
SGA	131 (59.8)
Sepsis	6 (2.7)
Presumed sepsis	6 (2.7)
Neonatal jaundice	12 (5.5)
Fever with rashes	2 (0.9)
IUGR	2 (0.9)
Premature	3 (1.4)

Note: SGA = small for gestation age; IUGR = intrauterine growth restriction

**Table 2:** Clinical manifestations and interpretation of congenital toxoplasmosis

Clinical presentation	No serological evidence *n* (%)	Passive immunity from mother *n* (%)	*P*-value
SGA			
Yes	70 (68.0)	61 (52.6)	0.021^a^
No	33 (32.0)	55 (47.4)	
Sepsis			
Yes	2 (1.9)	4 (3.4)	0.687^b^
No	101 (98.1)	112 (96.6)	
Presumed sepsis			
Yes	4 (3.9)	2 (1.7)	0.424^b^
No	99 (96.1)	114 (98.3)	
Neonatal jaundice			
Yes	7 (6.8)	5 (4.3)	0.420^a^
No	96 (93.2)	111 (95.7)	
Fever with rashes			
Yes	0 (0.0)	2 (1.7)	0.499^b^
No	103 (100.0)	114 (98.3)	
IUGR			
Yes	2 (1.9)	0 (0.0)	0.220^b^
No	101 (98.1)	116 (100.0)	
Premature			
Yes	0 (0.0)	3 (2.6)	0.249^b^
No	103 (100.0)	113 (97.4)	

Note:

a Pearson’s chi-squared test;

b Fisher’s exact test

**Table 3:** Clinical manifestations and interpretation of cCMV infection

Clinical presentation	No serological evidence *n* (%)	Acute infection *n* (%)	Passive immunity from mother *n* (%)	*P*-value
SGA				
Yes	1 (33.3)	2 (50.0)	128 (60.4)	0.597^a^
No	2 (66.7)	2 (50.0)	84 (39.6)	
Sepsis				
Yes	0 (0.0)	0 (0.0)	6 (2.8)	1.000^a^
No	3 (100.0)	4 (100.0)	206 (97.2)	
Presumed sepsis				
Yes	0 (0.0)	0 (0.0)	6 (2.8)	1.000^a^
No	3 (100.0)	4 (100.0)	206 (97.2)	
Neonatal jaundice				
Yes	0 (0.0)	1 (25.0)	11 (5.2)	0.330^a^
No	3 (100.0)	3 (75.0)	201 (94.8)	
Fever with rashes				
Yes	0 (0.0)	0 (0.0)	2 (0.9)	1.000^a^
No	3 (100.0)	4 (100.0)	210 (99.1)	
IUGR				
Yes	0 (0.0)	1 (25.0)	1 (0.5)	0.063^a^
No	3 (100.0)	3 (75.0)	211 (99.5)	
Premature				
Yes	0 (0.0)	0 (0.0)	3 (1.4)	1.000^a^
No	3 (100.0)	4 (100.0)	209 (98.6)	

Note:

a Fisher’s exact test;

IUGR = intrauterine growth restriction
